# Assessing Drug-Drug Interaction Potential among Patients Admitted to Surgery Departments in Three Palestinian Hospitals

**DOI:** 10.1155/2020/9634934

**Published:** 2020-09-23

**Authors:** Abdullah K. Rabba, Ayeshe M. Abu Hussein, Bayan K. Abu Sbeih, Somaya I. Nasser

**Affiliations:** Department of Pharmacy, Faculty of Pharmacy, Nursing, And Health Professions, Birzeit University, Birzeit, State of Palestine

## Abstract

**Background:**

Drug-drug interactions (DDIs) are a common issue that leads to adverse drug reactions in hospitals. Patients in the surgical department are expected to have potential DDIs that may lead to morbidity and mortality.

**Objectives:**

To study potential DDI prevalence in the surgery departments in 3 hospitals in Palestine. Moreover, to identify pertinent factors that are associated with drug-drug interactions.

**Method:**

A cross-sectional study in 3 governmental Palestinian hospitals: Palestine Medical Complex, Rafidia Hospital, and Beit Jala Hospital. Patients who are 20 years old or above and admitted to the surgical wards between September 2017 and February 2018 were included in the study. Patient demographics, all medications given in the hospital, and hospitalization period were obtained from medical files. The digital clinical decision support system Micromedex® was used for analysis and classification of possible drug interactions. Bivariate analysis and logistic regression were used to study the risk factors for developing DDIs.

**Results:**

502 patients were included in this report. The prevalence of potential DDIs among patients admitted to surgery wards in three Palestinian hospitals was 56%. The number of detected potential DDIs per patient was 2.22 ± 3.76. The number of prescribed medications (*P* < 0.001) was found to increase the possibility of having drug interactions.

**Conclusions:**

DDIs in Palestinian hospitals are a prevalent problem, and caution should be taken when ordering medications to hospitalized patients in surgery departments.

## 1. Introduction

The interactions between prescribed medications are a major and serious health issue that is facing the health care practice [1]. Drug-drug interaction (DDI) takes place when the effect of one drug is altered by a coadministered drug leading to harmful consequences that result from synergistic, additive, or antagonist effects [2]. Potential drug-drug interaction “is the possibility of one drug to interact with another when they are administered together” [3].

DDIs may be categorized into two major types: pharmacokinetic and pharmacodynamic drug interactions [2]. Kinetic drug interactions are those that affect the absorption, distribution, metabolism, and excretion of the drug, while dynamic drug interactions refer to the interactions that lead to interference with the physiological and therapeutic effects of the drug [4].

The harmful consequences that result from DDIs may be caused either by increasing the toxicity of the drug or by reduction in the efficacy of the drug [2]. Not all cases of drug interactions result in harmful consequences; in some cases, the interactions may be beneficial [2]. Drug interactions can also refer to situations such as physicochemical incompatibilities when mixing drug formulations together [2].

The possibility to have DDIs is known to be directly related to factors such as the age of the patient, number of prescribed drugs, and length of hospitalization [1].

Egger et al. demonstrated that elderly patients with ages ≥ 75 years are at greater risk of having potential DDIs compared to younger patients, since a higher number of drugs are prescribed to them, especially those that are used in treating chronic conditions, such as heart failure and arrhythmias [5].

Length of hospital stay was also shown to be an associated factor, since long hospitalization periods are related to more significant association with occurrence of DDIs [1, 6].

The overall effects and consequences of DDIs can lead to increase hospitalization period and cost [1].

According to a study carried out in Mexico City, 80.0% of ambulatory patients were found to have prescriptions with one or more potential DDIs and about 3.8% of patients were prescribed drugs with combinations that should be avoided due to interactions [7].

In another study carried out in Canada's largest cancer center in Toronto among cancer patients, Riechelmann et al. revealed that there was 276 potential DDIs among 405 patients; 9% were categorized as major interactions and 77% as moderate [8]. Most of these interactions were observed between nonanticancer medications such as antihypertensive agents and anticonvulsants [8]. Another study done in a Brazilian teaching hospital showed that the overall frequency of potential DDIs was about 49.7% [9]. 3.4% of these interactions were categorized as major, with hydrochlorothiazide and digoxin as the most interacting drugs [9]. According to a study conducted in Funen, Denmark, involving patients exposed to polypharmacy, it was observed that during one year, there was 6% of patients exposed to potential drug interaction [10].

According to a study done among Palestinian hemodialysis patients for evaluation of potential DDIs, 89.1% of patients had identified potential interactions; the most common interactions were calcium carbonate/amlodipine and calcium carbonate/aspirin [11]. Gout, hypertension, and diabetes were common comorbidities the study patients had, and the study showed that the association was significant between the total number of the medications prescribed for the patient and the number of potential DDIs [11].

The digital clinical decision support system Micromedex® is a tool that checks for potential drug interactions and gives a possible mechanism to explain the interaction and also provides information on the clinical management of the interaction [12]. Micromedex® was shown to be a reliable tool for drug interaction detection with high sensitivity and specificity [13, 14].

Micromedex® classifies drug-drug interactions according to onset, severity, and documentation [12].

Onset of drug interactions may be rapid in which the effect appears within 24 hours of administration, or it can be delayed, which occurs after 24 hours of administration [12]. According to severity, Micromedex classifies drug-drug interactions into major (which is life threatening and requires medical intervention), moderate (may require medical intervention), or minor (has a mild effect and often does not require medical intervention) [15–17]. With regard to documentation, Micromedex classifies interactions into excellent, good, fair, poor, or unlikely. Excellent documentation is associated with controlled clinical trials [15]. Good documentation refers to interactions evidenced by studies other than well controlled trials; fair and poor documentation are associated with interactions that lack good evidence to support them. Unlikely documentation lacks pharmacological basis [15].

Patients admitted to surgery departments are expected to be exposed to medications with the potential to interact with their prescribed medications or medications used to control their chronic conditions. Among the commonly used medications in surgery departments are antibiotics, analgesics, and CNS depressants. Thus, pDDIs among patients in surgery departments may have unique interactions compared to patients hospitalized in other hospital departments. Little is known about pDDIs in surgery departments [18, 19]. In addition, data regarding drug interaction potential in Palestine hospitals is scarce [11].

This study is aimed at assessing the drug interaction potential among patients admitted to surgery wards in Palestine hospitals. To our best knowledge, there are no published studies that address drug interactions in surgery wards in Palestine.

## 2. Method

### 2.1. Study Design and Population

This report is cross-sectional. It recruits patients admitted to surgical departments in three Palestinian hospitals in the West Bank, Palestine, between September 2017 and February 2018. The hospitals were Rafidia Hospital in Nablus city, Beit Jala Hospital in Bethlehem city, and Palestine Medical Complex in Ramallah city. These 3 governmental hospitals are the 3 main hospitals in the 3 main cities in the north, middle, and south of the West Bank, in Palestine. They have the major surgery departments in these 3 cities admitting patients with surgical complications and/or for surgical operations. All adult patients (≥20 years) admitted to the surgical department in the included hospitals during study period were taken in this study.

### 2.2. Sample Size

According to hospital records in the three hospitals where this study is carried out (nonpublished data), the total number of patients admitted to surgery departments in the three hospitals in the study period was 20000. An online sample size calculator (https://www.surveysystem.com/sscalc.htm) was used to calculate the required sample size for this study using a confidence level of 95% and it was 377. The sample size was increased by 30% to 35% so as to increase result reliability.

### 2.3. Data Source

Patients' electronic medical files were reviewed. Age, sex, previous medications, social history, all prescribed medications during hospitalization, dosage regimens, and hospitalization duration were collected from patients' files. The collected data was saved using SPSS software for future analysis.

### 2.4. Drug Interaction Analysis

For all patients included in this study, all prescribed medications were input into the digital clinical decision support system Micromedex® for the purpose of screening for potential DDIs. Each interaction was classified according to severity to contraindicated, major, moderate, or minor interaction.

### 2.5. Variables and Statistical Analysis

Statistical Package for the Social Sciences (SPSS) was used. Frequencies and percentages were presented for all variables. Mean and standard deviation or median were also given for continuous data. Bivariate analysis using a chi-square test was carried out to test for the association between categorical variables. Risk factors associated with *P* values less than 0.1 in the bivariate analysis were studied using a multiple binary logistic regression model where the dependent variable was having or not having a potential drug interaction. *P* values less than 0.05 were considered to be statistically significant.

## 3. Results

502 patients who were admitted to surgery departments were included in the study. The mean age of patients was 49.5 ± 17.8 with a minimum age of 20 years and a maximum of 90. 53.8% of patients were males and 46.2% were females. Age and sex categories are shown in Table 1.

Of the total number of patients, 281 patients (56%) had at least one potential drug-drug interaction (pDDI). And 221 patients (44%) had no interactions.

A total of 1114 drug-drug interactions were identified and classified according to severity to major, moderate, minor, and contraindicated interactions; their frequency and percentages are shown in Table 2. Descriptive statistics of detected pDDIs among study participants is presented in Table 3 where the number of potential DDIs detected per patient was 2.22 ± 3.76.

Table 4 presents the bivariate analysis of factors that may interfere with having pDDIs. These factors include age, number of prescribed drugs, and length of hospital stay.

In the multiple binary logistic regression model, “having” or “not having” a pDDI is the dependent variable and age, number of medications, and duration of hospital stay are the predictor variables. The potential for drug interaction significantly increased with the number of prescribed drugs. The results of the logistic regression model analysis are shown in Table 5.

Common pairs of coprescribed medications with detected pDDI are presented in Table 6; the most common coprescribed medications with significant drug-drug interaction potential were ranitidine+meperidine and bisoprolol+aspirin; they were detected in 14.2% and 7.3% of drug interactions, respectively.

## 4. Discussion

In this study, the mean of prescribed drugs for patients was 7.83 ± 4.63. This is similar to a Palestinian study that evaluated potential DDIs in hemodialysis patients where the mean was 7.87 ± 2.44 [11]. Results in this report are slightly higher than the mean reported in a study in a medical ward of a teaching hospital in India where it was 6 ± 2.13 [16]. However, a study conducted in a teaching hospital in Iran reported a slightly higher mean of prescribed drugs (9.1 ± 4.3) [17]. These differences may be due to different clinical practice in different countries and/or different hospital settings, since majority of these studies were not conducted in surgical departments and the difference could be due to different drugs given in each ward of the hospital.

The prevalence of pDDIs in this study was 56%. According to the severity, most of these interactions were classified as major interactions (52.7%), followed by moderate (40.5%), then minor (6.4%) and contraindicated interactions (0.4%). This is higher than the percentage reported in a study done in a surgical ward in a Mexican hospital which was 49.5% [18]. This difference may be due to the difference of the methodology used for identification and classification of pDDIs. Other studies in surgical departments are few. A Brazilian study of drug interactions in the ICU reported a higher prevalence of pDDIs which was 89% [19].

The most frequently encountered DDI in this study in 3 Palestinian surgery departments was the meperidine+ranitidine combination. This interaction that involves the opioid analgesic, meperidine, may be one of the interactions that have a high risk of taking place in surgery departments, since analgesics are among the commonly used medications in surgery departments. In a Mexican surgery department [18], metronidazole and fluoroquinolone were among the most frequently interacting drugs; the use of these drugs is also common in surgery departments. Compared to our study, metronidazole and fluoroquinolone interactions were more frequent.

Age of patients is expected to be associated with higher risk of interactions possibly due to higher risk of having comorbid conditions.

Long hospitalization periods are possibly associated with having potential DDIs, since the patients are expected to have more new prescribed drugs during the hospital stay period [6].

Among the detected drug interactions that need be taken into account is ciprofloxacin and metronidazole; these drugs cause QT prolongation and their concurrent use increases the risk of this side effect, and so attention should be given to such interaction [20].

In addition, aspirin and furosemide are a risky combination; aspirin decreases the diuretic action of furosemide by the inhibition of renal prostaglandin synthesis [21].

Warfarin and acetaminophen also have the potential to interact; it is not well understood how acetaminophen increases the warfarin effect [22]. One possible mechanism is that acetaminophen inhibits the enzymes that are involved in the vitamin K-dependent coagulation factor synthesis [23]. Clinicians should give attention towards this clinical interaction. Four grams of acetaminophen per day or higher can potentiate the anticoagulant response [23].

Furthermore, clopidogrel and atorvastatin are a combination that needs caution. Clopidogrel is activated by CYP3A4, and atorvastatin is another CYP3A4 substrate; due to competition over CYP3A4, atorvastatin inhibits clopidogrel activation. Other statins that are not metabolized by CYP3A4 can be used instead [24].

Meperidine and ranitidine are also of interest. Meperidine is metabolized by N-demethylation by CYP3A4 to normeperidine [25] and ranitidine is a CYP3A4 inhibitor, but the effect has little clinical significance [26].

Aspirin and ranitidine also have potential interaction where ranitidine can alter the kinetics of aspirin. A single dose of aspirin does not cause clinically significant alteration [27]. Ranitidine can lead to modest decrease in the effect of aspirin (platelet aggregation); the most possible mechanism for this interaction is that ranitidine can change the absorption conditions of aspirin [28].

The exact mechanism for the reaction of bisoprolol fumarate and aspirin is not well known; it seems that aspirin decreases the vasodilation mediated by beta blockers in patients with hypertension and heart failure [29].

The limitations of this study include not studying the effect of surgical history and comorbid conditions on the development of DDIs and not assessing the clinical significance of DDIs in patients.

## 5. Conclusion

Drug-drug interaction in this study is shown to be a highly prevalent issue among patients admitted to surgery departments in Palestine. Caution should be taken when prescribing and administering drugs to hospitalized patients. The number of prescribed drugs needs to be considered when prescribing medications to patients admitted to surgery wards. Antibiotics (e.g., metronidazole and ciprofloxacin) and analgesics (e.g., meperidine and acetaminophen) are among the drugs that can possibly result in common DDIs in surgery patients.

## Figures and Tables

**Table 1:** Sex and age distribution among patients admitted to surgery wards in 3 Palestinian hospitals (*n* = 502).

		Frequency	Percent
Sex	Male	270	53.8%
	Female	232	46.2%
Age (years)	20-39	156	31.1%
	40-59	199	39.6%
	60-79	116	23.1%
	≥80	31	6.2%

**Table 2:** Type of drug interaction detected among 502 patients admitted to surgery wards in 3 Palestinian hospitals (total number of detected interactions is 1114).

Severity of drug interaction	Frequency	Percentage
Major	587	52.7%
Moderate	451	40.5%
Minor	71	6.4%
Contraindication	5	0.5%
Total	1114	100%

**Table 3:** Descriptive statistics of potential drug interaction detected in 502 patients admitted to surgery wards in 3 Palestinian hospitals (total number of detected interactions is 1114).

Variable	Minimum	Maximum	Median	Mean	Standard deviation
Number of prescribed drugs per patient	2	32	5	7.83	4.63
Number of potential DDIs per patient	0	35	1	2.22	3.76

**Table 4:** Bivariate analysis of factors associated with potential drug-drug interactions among 502 patients admitted to surgery wards in 3 Palestinian hospitals.

Factor		Presence of DDIs (*n* (%))	Absence of DDIs (*n* (%))	*P* value
Sex	Males	154 (57.0%)	116 (43.0%)	0.605
	Females	127 (54.7%)	105 (45.3%)	
Age (years)	20-39	76 (48.7%)	80 (51.3%)	0.077
	40-59	112 (56.3%)	87 (43.7%)	
	60-79	72 (62.0%)	44 (38.0%)	
	≥80	21 (67.7%)	10 (32.3%)	
Number of prescribed drugs	2-4	21 (19.4%)	87 (80.6%)	<0.001
	5-7	91 (47.4%)	101 (52.6%)	
	8-10	72 (74.2%)	25 (25.8%)	
	≥11	97 (93.3%)	7 (6.7%)	
Hospitalization period (days)	1-3	88 (49.2%)	91 (50.8%)	0.072
	4-6	113 (59.5%)	77 (40.5%)	
	≥7	80 (60.2%)	53 (39.8%)	

**Table 5:** Multiple binary logistic regression model for factors associated with potential drug-drug interactions among 502 patients admitted to surgery wards in 3 Palestinian hospitals.

Predictor variable		*β* coefficient of predictor variables	Standard error	Wald	*P* value	Odds ratio (95% CI)
Age (years)	20-39			0.89	0.83	Reference
	40-59	0.00	0.25	0.00	0.99	1.00 (0.617-1.631)
	60-79	-0.24	0.30	0.62	0.43	0.79 (0.43-1.43)
	≥80	-0.20	0.49	0.17	0.68	0.82 (0.31-2.14)
Number of prescribed drugs	2-4			96.78	0.00	Reference
	5-7	1.31	0.29	21.13	0.00	3.70 (2.12-6.47)
	8-10	2.49	0.34	53.30	0.00	12.03 (6.17-23.46)
	≥11	4.12	0.47	75.91	0.00	61.40 (24.32-155.02)
Hospitalization period (days)	1-3			2.99	0.23	Reference
	4-6	0.33	0.25	1.74	0.19	1.39 (0.85-2.26)
	≥7	0.43	0.27	2.57	0.11	1.54 (0.91-2.63)

**Table 6:** Common interactions among 502 patients admitted to surgery wards in 3 Palestinian hospitals.

Interacted pairs	*N* (% per total number of detected interactions)	Severity	Effect	Possible mechanism
Ranitidine HCl+meperidine HCl	158 (14.2%)	Major	Prolongation of opioid effects	Inhibition of meperidine metabolism
Bisoprolol fumarate+aspirin	81 (7.3%)	Moderate	Increased blood pressure	Decreased production of renal prostaglandins
Aspirin+furosemide	66 (5.9%)	Major	Reduced diuretic effectiveness and possible nephrotoxicity	Decreased renal prostaglandin synthesis
Clopidogrel+atorvastatin calcium	60 (5.4%)	Moderate	High on-treatment platelet reactivity	Competition with CYP3A4-mediated clopidogrel metabolism
Ciprofloxacin+metronidazole	54 (4.8%)	Major	QT prolongation and arrhythmias	QT prolongation
Warfarin sodium+acetaminophen	51 (4.6%)	Moderate	Increased risk of bleeding	Inhibition of warfarin metabolism
Ranitidine hydrochloride+aspirin	31 (2.8%)	Minor	Reduced salicylate plasma levels and decreased antiplatelet effect of aspirin	Reduced absorption of aspirin
Phenytoin+ranitidine hydrochloride	20 (1.8%)	Minor	Increased phenytoin concentrations	Decreased phenytoin metabolism

## Data Availability

The datasets analyzed during the current study is available from the corresponding author on reasonable request.

## References

[1] Moura C. S., Acurcio F. A., Belo N. O. (2009). Drug-drug interactions associated with length of stay and cost of hospitalization. *Journal of Pharmacy & Pharmaceutical Sciences*.

[2] Baxter K. (2008). Stockley’s drug interactions. *Stock Drug Interact*.

[3] Morales-Ríos O., Jasso-Gutiérrez L., Reyes-López A., Garduño-Espinosa J., Muñoz-Hernández O. (2018). Potential drug-drug interactions and their risk factors in pediatric patients admitted to the emergency department of a tertiary care hospital in Mexico. *PLoS One*.

[4] Sousa M., Pozniak A., Boffito M. (2008). Pharmacokinetics and pharmacodynamics of drug interactions involving rifampicin, rifabutin and antimalarial drugs. *The Journal of Antimicrobial Chemotherapy*.

[5] Egger S. S., R??tz Bravo A. E., Hess L., Schlienger R. G., Kr??henb??hl S. (2007). Age-related differences in the prevalence of potential drug-drug interactions in ambulatory dyslipidaemic patients treated with statins. *Drugs & Aging*.

[6] Murtaza G., Khan M. Y. G., Azhar S., Khan S. A., Khan T. M. (2016). Assessment of potential drug-drug interactions and its associated factors in the hospitalized cardiac patients. *Saudi Pharmaceutical Journal*.

[7] Doubova S. V., Reyes-Morales H., Torres-Arreola L. P., Suárez-Ortega M. (2007). Potential drug-drug and drug-disease interactions in prescriptions for ambulatory patients over 50 years of age in family medicine clinics in Mexico City. *BMC Health Services Research*.

[8] Riechelmann R. P., Tannock I. F., Wang L., Saad E. D., Taback N. A., Krzyzanowska M. K. (2007). Potential drug interactions and duplicate prescriptions among cancer patients. *Journal of the National Cancer Institute*.

[9] Cruciol-Souza J. M., Thomson J. C. (2006). Prevalence of potential drug-drug interactions and its associated factors in a Brazilian teaching hospital. *Journal of Pharmacy & Pharmaceutical Sciences*.

[10] Bjerrum L., Gonzalez Lopez-Valcarcel B., Petersen G. (2008). Risk factors for potential drug interactions in general practice. *The European Journal of General Practice*.

[11] al-Ramahi R.’., Raddad A. R., Rashed A. O. (2016). Evaluation of potential drug- drug interactions among Palestinian hemodialysis patients. *BMC Nephrology*.

[12] Bista D., Saha A., Mishra P., Palaian S., Shankar P. R. (2009). Impact of educational intervention on the pattern and incidence of potential drug-drug interactions in Nepal. *Pharmacy practice*.

[13] Kheshti R., Aalipour M., Namazi S. (2016). A comparison of five common drug–drug interaction software programs regarding accuracy and comprehensiveness. *Journal of research in pharmacy practice*.

[14] Roblek T., Vaupotic T., Mrhar A., Lainscak M. (2015). Drug-drug interaction software in clinical practice: a systematic review. *European Journal of Clinical Pharmacology*.

[15] Sharma S., Chhetri H., Alam K. (2014). A study of potential drug-drug interactions among hospitalized cardiac patients in a teaching hospital in Western Nepal. *Indian journal of pharmacology*.

[16] Ahmad A., Khan M. U., Haque I. (2015). Evaluation of potential Drug - Drug interactions in general medicine ward of teaching hospital in Southern India. *Journal of clinical and diagnostic research: JCDR*.

[17] Mousavi S., Ghanbari G. (2017). Potential drug-drug interactions among hospitalized patients in a developing country. *Caspian journal of internal medicine*.

[18] Sánchez-López V. A., Brennan-Bourdon L. M., Rincón-Sánchez A. R., Islas-Carbajal M. C., Navarro-Ruíz A., Huerta-Olvera S. G. (2016). Prevalence of potential drug-drug interactions in hospitalized surgical patients. *The Journal of Pharmacy and Pharmacology*.

[19] Rodrigues A. T., Stahlschmidt R., Granja S., Pilger D., Falcão A. L. E., Mazzola P. G. (2017). Prevalence of potential drug-drug interactions in the intensive care unit of a Brazilian teaching hospital. *Brazilian Journal of Pharmaceutical Sciences*.

[20] Khan Q., Ismail M., Khan S. (2017). Frequency, characteristics and risk factors of QT interval prolonging drugs and drug-drug interactions in cancer patients: a multicenter study. *BMC Pharmacology and Toxicology*.

[21] Bartoli E., Arras S., Faedda R., Soggia G., Satta A., Olmeo N. A. (1980). Blunting of furosemide diuresis by aspirin in man. *Journal of Clinical Pharmacology*.

[22] Hylek E. M., Heiman H., Skates S. J., Sheehan M. A., Singer D. E. (1998). Acetaminophen and other risk factors for excessive warfarin anticoagulation. *JAMA*.

[23] Mahé I., Bertrand N., Drouet L. (2006). Interaction between paracetamol and warfarin in patients: a double-blind, placebo-controlled, randomized study. *Haematologica*.

[24] Lau W. C., Waskell L. A., Watkins P. B. (2003). Atorvastatin reduces the ability of clopidogrel to inhibit platelet aggregation: a new drug-drug interaction. *Circulation*.

[25] Buck M. L. (2011). Is meperidine the drug that just won’t die?. *Journal of Pediatric Pharmacology and Therapeutics*.

[26] Martinez C., Albet C., Agundez J. (1999). Comparative in vitro and in vivo inhibition of cytochrome P450 CYP1A2, CYP2D6, and CYP3A by H-receptor antagonists. *Clinical Pharmacology and Therapeutics*.

[27] Corrocher R., Bambara L. M., Caramaschi P. (1987). Effect of ranitidine on the absorption of aspirin. *Digestion*.

[28] Lev E. I., Ramabadran R. S., Guthikonda S. (2007). Effect of *ranitidine* on the antiplatelet effects of *aspirin* in healthy human subjects. *The American Journal of Cardiology*.

[29] Lindenfeld J., Robertson A. D., Lowes B. D., Bristow M. R., MOCHA (Multicenter Oral Carvedilol Heart failure Assessment) Investigators (2001). aspirin impairs reverse myocardial remodeling in patients with heart failure treated with beta-blockers. *Journal of the American College of Cardiology*.

